# Intercellular transfer of P-glycoprotein in human blood-brain barrier endothelial cells is increased by histone deacetylase inhibitors

**DOI:** 10.1038/srep29253

**Published:** 2016-07-04

**Authors:** Andreas Noack, Sandra Noack, Manuela Buettner, Hassan Y. Naim, Wolfgang Löscher

**Affiliations:** 1 From the Department of Pharmacology, Toxicology, and Pharmacy, University of Veterinary Medicine Hannover, Germany; 2 Department of Trauma Surgery, Hannover Medical School, Germany; 3 Institute of Laboratory Animal Science, Hannover Medical School, Germany; 4 Department of Physiological Chemistry, University of Veterinary Medicine Hannover, Germany; 5 Center for Systems Neuroscience, Hannover, Germany

## Abstract

The blood–brain barrier (BBB) controls the entry of compounds into the brain, thereby regulating brain homeostasis. Efflux transporters such as P-glycoprotein (Pgp) significantly contribute to BBB function. Multiple signaling pathways modulate the expression and activity of Pgp in response to xenobiotics and disease. A non-genetic way of intercellular transfer of Pgp occurs in cancer cells, but whether this also occurs in non-cancer cells such as endothelial cells that form the BBB is not known. A human brain endothelial cell line (hCMEC/D3) was used to study whether cell-to-cell Pgp transfer occurs during co-culturing with Pgp-EGFP expressing hCMEC/D3 cells. The Pgp-EGFP fusion protein was transferred from donor to recipient cells by cell-to-cell contact and Pgp-EGFP enriched vesicles, which were exocytosed by donor cells and endocytosed by adherent recipient cells. Flow cytometry experiments with the Pgp substrate eFLUXX-ID Gold demonstrated that the transferred Pgp is functional in the recipient cells. Exposure of the donor cells with inhibitors of histone deacetylases (HDACs) resulted in an enhanced intercellular Pgp transfer. Non-genetic transfer of a resistance phenotype and its regulation by HDACs is a novel mechanism of altering BBB functionality. This mechanism may have important implications for understanding drug-induced alterations in Pgp expression and activity.

Intercellular transfer of proteins is an integral part of communication between cells, involving mechanisms such as tunneling nanotubes bridging neighboring cells or release and binding of protein-containing membrane microparticles and extracellular vesicles^1^. In 2005, Levchenko *et al.*^2^ reported that intercellular transfer of the efflux transporter P-glycoprotein (Pgp; MDR1; ABCB1) mediates acquired multidrug resistance in tumor cells. This report added another dimension to the ways cells can acquire a particular cell surface protein-mediated phenotype such as multidrug resistance^1^. Since then, numerous studies have confirmed the finding of Levchenko *et al.*^2^, showed that intercellular Pgp transfer occurs in different cancer cell lines, and explored mechanisms involved in this transfer^3^,^4^,^5^,^6^,^7^,^8^,^9^,^10^,^11^,^12^,^13^. However, drug resistance is not only a problem in cancer treatment, but also several other diseases, including brain disorders such as epilepsy or depression^14^,^15^,^16^,^17^. Overexpression of Pgp at the blood-brain barrier (BBB) is discussed as a major mechanism of pharmacoresistance in such disorders, but the exact mechanisms of the overexpression are only incompletely understood. Both intrinsic (polymorphisms in the Pgp-coding gene *ABCB1*) and acquired (induction of *ABCB1* expression by seizures or drug treatment) mechanisms are discussed^14^,^15^,^16^,^18^,^19^,^20^.

In the present study, we investigated whether intercellular Pgp transfer as reported for cancer cells is also a physiological defense mechanism of brain capillary endothelial cells that form the BBB. By using human brain capillary endothelial cells (hCMEC/D3) that were stably transfected with a doxycycline-inducible MDR1-EGFP fusion plasmid, we have recently shown drug-induced intracellular trafficking of Pgp^21^, but it is not known whether intercellular trafficking occurs at the BBB and can enhance drug efflux. By using hCMEC/D3-MDR1-EGFP cells (Pgp-donor cells) co-cultured with hCMEC/D3 wildtype cells (Pgp-recipient cells), we now demonstrate intercellular Pgp transfer and its functional relevance for the recipient cells, induction of this process by the major antiepileptic drug (AED) valproate, and possible involvement of inhibition of histone deacetylases (HDACs) in this drug effect. These findings have important implications for BBB functioning and resistance to therapy.

## Materials and Methods

### Cell culture conditions

Human brain endothelial cells (hCMEC/D3^22^) were kindly provided by Dr. Pierre-Olivier Couraud, Institut COCHIN, Paris, France. In addition, conditional doxycycline-inducible Pgp-EGFP and EGFP expressing hCMEC/D3 cells were produced as described previously in detail^21^. In co-culture experiments (see below), hCMEC/D3-MDR1-EGFP cells served as Pgp-donor cells while hCMEC/D3 cells served as Pgp-recipient (or “wildtype”) cells. Cells were cultivated in endothelial cell basal medium-2 (EBM-2, Lonza, Cologne, Germany) supplemented with 5% fetal calf serum (PAA Laboratories, Cölbe, Germany), 1% penicillin (100 U/ml), streptomycin (100 μg/ml) (Invitrogen, Karlsruhe, Germany), 1.4 μM hydrocortisone (Sigma-Aldrich, Munich, Germany), 5 μg/ml ascorbic acid (Sigma-Aldrich), 1% lipid concentrate (Invitrogen), 10 mM HEPES (Invitrogen) and 1 ng/ml basic FGF (Sigma-Aldrich).

### Pgp-EGFP transfer experiments

hCMEC/D3-MDR1-EGFP cells (1 × 10^5^; Pgp-donor cells) were co-cultured with wildtype hCMEC/D3 cells (1 × 10^5^; Pgp-recipient cells) in 6 well plates for 48 h. Before co-culturing, the hCMEC/D3 cells were labeled with CellTracker Red CMTPX (Life Technologies, Darmstadt, Germany) to enable the combination of the Pgp substrate eFLUXX-ID Gold (ENZO Life Sciences, Lörrach, Germany) with a cell labeling substance. eFLUXX-ID Gold has been optimized for multiplexing with other common fluorescent dyes in flow cytometric assays^23^, allowing the concomitant use of several dyes as done in this study. In this respect, the eFLUXX-ID Gold uptake assay has advantages compared to more commonly used Pgp substrates, such as rhodamine 123^23^. As rhodamine 123, eFluxx-ID Gold is not a selective Pgp substrate, but is also transported by multidrug resistance protein(MRP)-1 and breast cancer resistance protein^23^. By using specific inhibitors of these ABC transporters, the transporter involved in eFLUXX-ID Gold efflux can be specified^23^,^24^. The hydrophobic, non-fluorescent eFLUXX-ID Gold readily penetrates the cell membrane, and is hydrolyzed to a hydrophilic fluorescent dye by intracellular esterases. Unless the EFLUXX-ID dye is pumped out of the cell, the esterase cleaved dye is trapped inside the cell^23^. In several cell lines, the eFluxx-ID Gold probe has been shown to be more sensitive for Pgp activity detection than other commonly used probes^23^.

In addition to CellTracker Red CMTPX for labeling wildtype (hCMEC/D3) cells, Cell Proliferation Dye eFluor670 (eBioscience, Frankfurt, Germany) was used according to the manufacturer’s protocol. To induce Pgp-EGFP expression in the Pgp-donor cells, they were cultivated in cell culture media with 1 mg/ml doxycycline (Biochrom, Berlin, Germany), which leads to a massive overexpression of the transporter^21^.

In experiments with drug exposure, Pgp-donor cells were drug-exposed before co-cultivation as described below to ensure that the Pgp-recipient cells do not receive the respective treatment. Labeled Pgp-recipient cells were co-cultured with an equal number of Pgp-donor cells or, for control, hCMEC/D3-EGFP cells for 48 h.

### Drug experiments

To investigate whether drugs modulate intercellular Pgp transfer in human brain endothelial cells, Pgp-donor cells were exposed to the AEDs valproate (VPA) and phenobarbital (PB) three days after reaching confluence, immediately before the co-culture experiments with Pgp-recipient cells. Both drugs have previously been reported to induce Pgp expression in different cell types^25^,^26^, but not in hCMEC/D3 cells^27^. VPA exhibits diverse effects on cells, which include the inhibition of HDACs^28^. For comparison with VPA, we also tested the effects of the more specific HDAC-inhibitor trichostatin A (TSA). We also included the cytostatic Pgp-trafficking-inducer mitomycin C (MMC), which has been described by us previously to induce intracellular Pgp trafficking in human brain endothelial cells^21^. Cells were treated with 300 μM or 1 mM VPA, 100 μM PB^27^ or 330 nM or 1 μM TSA for 24 h. Drug concentrations for VPA and TSA were based on the ability to inhibit HDACs^29^,^30^. For MMC treatment cells were treated with 1 μM MMC for 4 h in Opti-MEM (Invitrogen). Subsequently, medium was removed after 4 hours of MMC and cells were treated with fresh culture medium as described before^21^. In order to exclude that the observed effects of HDAC inhibitors on intercellular Pgp transfer were due to increased Pgp expression, both Pgp-donor and -recipient cells were exposed to VPA and TSA for 24 h and Pgp expression was measured by Western blot (see below).

### Live cell imaging

**Figure 1:**
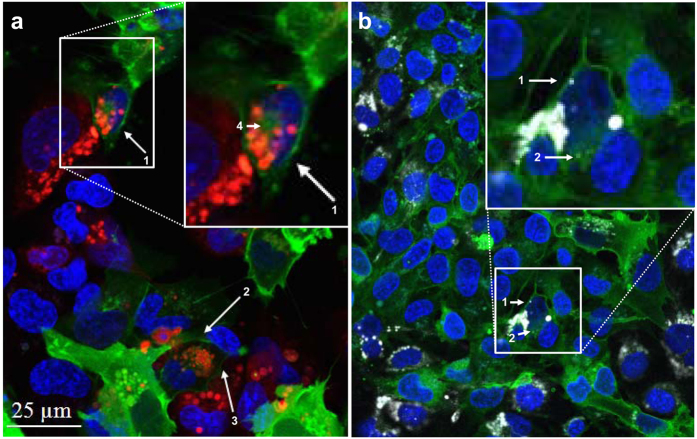
Intercellular Pgp-EGFP transfer. To investigate Pgp-EGFP transfer from hCMEC/D3-MDR1-EGFP cells (Pgp-donor cells) to hCMEC/D3 cells (Pgp-recipient cells) with confocal fluorescence microscopy, Pgp-recipient cells were labeled with CMTPX (**a**; red) or eFluor670 (**b**; white) and co-cultured with an equal number of Pgp-donor cells (green). Representative images are shown. Consistent with the seeding of cells, about half of the co-culture are either red (**a**) or white (**b**) labeled Pgp-recipient cells. (**a**) The arrows (1–4) show the fusion protein Pgp-EGFP (in green), which was transferred to red labeled Pgp-recipient cells. Image **a** indicates that the Pgp-transfer to brain endothelial cells occurs by cell-to-cell contact, as in a arrow 1, a red-labeled Pgp-recipient cells (middle of magnification) that is in contact with a Pgp-donor cell (top right of magnification) receives the fusion protein Pgp-EGFP, whereas another hCMEC/D3 cell (bottom left of magnification), which is not in direct contact with the Pgp-donor cell (top right of magnification), does not receive the fusion protein. (**b**) In another co-culture experiment, the cytotracker CMTPX was replaced by eFluor670 to allow the combination of a further fluorescent dye (eFLUXX-ID Gold, which was used as a Pgp substrate in subsequent flow cytrometric experiments) and to confirm the results shown in **a**. Consistent with the results in image **a**, the arrows in **b** show a transfer of Pgp-EGFP on eFluor 670 (white) labeled Pgp-recipient cell. Fluorescent images of the co-culture in **b** shows DNA in blue, hCMEC/D3 cells in white and fusion protein Pgp-EGFP in green (see Fig. S1 for further explanation). Interestingly, it was observed in **a** and **b** that the Pgp-EGFP fusion protein is transferred to neighboring cells. Direct cell-to-cell contact seems to be necessary, since the fusion protein Pgp-EGFP is only transferred on labeled Pgp-recipient cells, which are in direct contact to Pgp-donor cells (arrows in **a** and **b**). In **a** (arrows 1–3) and in **b** (arrow 1) transferred Pgp-EGFP in Pgp-recipient cells is localized to the membrane in a punctuate pattern and in **a** (arrow 4) and **b** (arrow 2) also intracellularly.

Live cell imaging analyses were performed by utilizing two different fluorescence microscope systems. Initial live cell imaging experiments as shown in Fig. 1 were performed on a confocal fluorescence microscopic system (Leica SP5, Leica Microsystems, Bensheim, Germany). Long term (48 hours) experiments as shown in Video S1 (see Supporting Information) were performed in an incubator with an inverted fluorescence microscope (Lumascope 500, Etaluma, Carlsbad, USA). Cells were co-cultured as described above. For confocal fluorescence microscopic experiments cells were plated on collagen type I (100 μg/ml; Invitrogen)-coated 100-mm tissue culture plates (Sarstedt, Nümbrecht, Germany) containing a 42-mm glass plate (H. Sauer Laborbedarf, Reutlingen, Germany). The nuclei of living cells were stained for 30 min with 5 mM bisbenzimide H (Sigma-Aldrich) at 37 °C in phenol red free Opti-MEM medium (Invitrogen). Glass plates with cells were implemented into a PeCon open chamber (PeCon, Erbach, Germany). Excitation wavelengths of 405 nm (bisbenzimide H), 481 nm (Pgp-EGFP), 561 nm (CMTPX, red) or 633 nm (eFluor670) were used. For 48 hours live cell imaging experiments, cells were co-cultured in an incubator with an inverted fluorescence microscope as described above. Images were taken every 10 minutes and excitation wavelengths of 488 nm were used.

### Flow cytometry of Pgp-EGFP transfer in co-cultured cells

The transfer of Pgp-EGFP from Pgp-donor to Pgp-recipient cells was measured by flow cytometric analysis. Pgp-donor cells were co-cultured with CMTPX or eFluor670 labeled Pgp-recipient cells as described above. To measure Pgp activity in co-cultures of Pgp-donor cells and eFluor670 labeled Pgp-recipient cells, cellular uptake of the Pgp substrate eFLUXX-ID Gold (ENZO Life Sciences) of untreated and Pgp inhibitor-treated (1 hour, 0.5 μM tariquidar or 20 μM verapamil) co-cultures was determined. For this, co-cultured cells were trypsinized and incubated for 30 min at 37 °C in phenol red free Opti-MEM medium (Invitrogen) with eFLUXX-ID Gold according to the manufacturer’s protocol. Unlabeled Pgp-recipient cells, CMTPX labeled recipient cells, eFluor670 and Pgp-donor cells were used as controls. Cells were measured with the parameters forward scatter (FSC) and side scatter (SSC), CMTPX or eFLUXX-ID Gold (PE channel), eFluor670 (APC channel) and EGFP (FITC channel). All experiments were performed in triplicate with the measurement of 10,000 individual cells.

For analysis of Pgp-EGFP activity, the multidrug resistance factor (MAF) described by Huber *et al.*^24^ was calculated by the following formula for each probe: MAF_Pgp_ = 100 × (mean fluorescence intensity (MFI) of tariquidar or verapamil treated cells – MFI of untreated cells)/MFI of tariquidar or verapamil treated cells.

### Western blot analysis

Detergent extracts of the HCMEC/D3 cells and hCMEC/D3-MDR1-EGFP cells were prepared as described before^21^ and subjected to Western blot analysis using anti-PGP 1:200 (Signet Laboratories, Dedham, MA, USA), anti-acetyl-histone-H4 1:200 (pan Lys 5, 8, 12, Merck Chemicals GmbH, Schwalbach, Deutschland) and anti-actin 1:1000 (Sigma-Aldrich) as primary antibiodies. The secondary antibodies utilized anti-mouse-HRP 1:1000 and anti-rabbit-HRP 1:1000 (Dako, Hamburg, Germany). Proteins were visualized by enhanced chemiluminescence using SuperSignal West Femto Chemiluminescent Substrate (Thermo Scientific) and the ChemiDoc system (Bio-Rad, Munich, Germany) with QuantityOne software (Bio-Rad) according to the manufacturer’s instructions. The indicated protein signals were quantified densitometrically with QuantityOne software (Bio-Rad) and calculated by normalization to the reference signals of actin with GraphPad Prism software (GraphPad, San Diego, CA, USA).

### Statistics

All data are expressed as the mean ± SEM of three or more experiments. The significance of intergroup differences was calculated using either Student’s t-test or, when data were not normally distributed, the U-test of Mann and Whitney. Deviations from Gaussian distribution were determined using the Kolmogorov-Smirnov test. For analysis of differences between several groups in the same experiment, analysis of variance (ANOVA) followed by Dunnett’s or Bonferroni’s Multiple Comparison Tests were used. Tests used were two-sided and a P < 0.05 was considered significant. All statistical analyses were performed by the PRISM 6 software (GraphPad Software Inc., La Jolla, CA, USA).

## Results

### Intercellular Pgp-transfer between adherent human brain capillary endothelial cells

To study whether Pgp-EGFP-transfer occurs in human brain capillary endothelial cells, hCMEC/D3 cells (Pgp-recipient cells) were co-cultured with an equal number of hCMEC/D3-MDR1-EGFP cells (Pgp-donor cells). The recipient cells were labelled before seeding with fluorescent dye CellTracker™ Red CMTPX or eFluor670 to permit their identification in the co-cultures by confocal microscopy. In both cases equivalent cell numbers were co-cultured with Pgp-donor cells. As shown in Fig. 1 and Video S1, such co-cultures in combination with confocal microscopy and flow cytometry allow monitoring the trafficking of the Pgp-EGFP fusions protein in a monolayer of human brain endothelial cells.

Figure 1 illustrates intercellular Pgp-EGFP transfer from the donor to the recipient cells, most probably by direct cell-to-cell contact between adherent cells. The Pgp-EGFP fusion protein was revealed as punctuated structures at the cell membrane of labeled recipient hCMEC/D3 cells. The localization of the transferred fusion protein Pgp-EGFP at the membrane of the Pgp-recipient cells suggested that the multidrug resistance efflux pump is functional in these cells. Moreover the fusion protein Pgp-EGFP was localized intracellularly in labeled recipient cells, which is a further indication that the fusion protein Pgp-EGFP might be functional, since it was transported intracellularly in labelled recipient cells most likely by endocytosis mechanisms (Fig. 1). As shown in Fig. S1, the fluorescent CellTracker was not transferred between cells.

The mechanism of Pgp-EGFP transfer was further evaluated in live images of co-cultured cells that were incubated for 48 hours. Strikingly, Pgp-EGFP was transferred in Pgp-EGFP enriched vesicles that were exocytosed by Pgp-EGFP donor cells and endocytosed by adherent P-gp-EGFP recipient cells or were exocytosed to the supernatant of human brain endothelial cells (Video S1; arrows).

**Figure 2:**
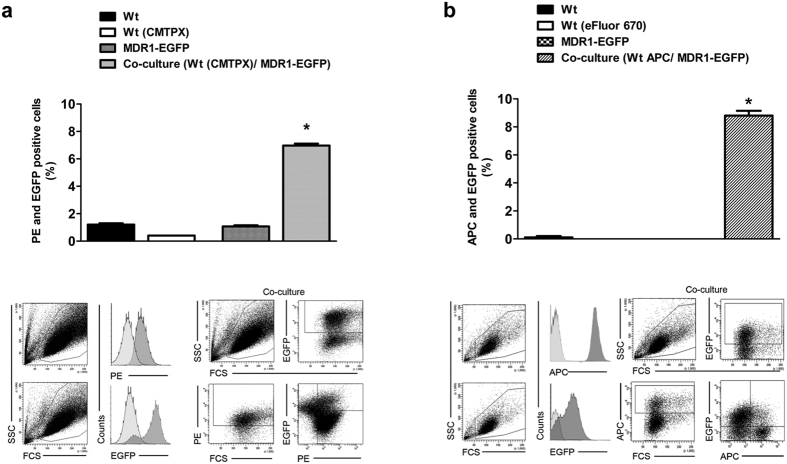
Flow cytometric detection of intercellular Pgp-EGFP. Transfer of Pgp-EGFP between hCMEC/D3-MDR1-EGFP (Pgp-donor) and hCMEC/D3 (Pgp-recipient) cells was measured quantitatively by flow cytometry after 48 hours of co-cultivation. Therefore Pgp-donor cells were co-cultured with an equal amount of CMTPX (**a**) or eFluor670 (**b**) labeled Pgp-recipient cells. The extent of Pgp-EGFP transfer to the recipient cells was measured by flow cytometry of double positive PE (**a**, CMTPX) or APC (**b**, eFluor 670), and EGFP (**a,b**) cells. To control the double positive parameters, single cultures of unlabeled hCMEC/D3 wildtype cells, CMTPX labeled hCMEC/D3 wildtype cells (**a**), eFluor670 (**b**) and hCMEC/D3-MDR1-EGFP cells were used. Both experimental setups showed a similar extent of Pgp-EGFP transfers. The experiments were performed in triplicate with the measurement of 10,000 individual cells. Significant differences of PE (**a**) or APC (**b**) and EGFP positive cells versus control cells are indicated by asterisk (P < 0.05).

For quantification of the increased amount of Pgp-EGFP in labelled recipient cells, the co-cultured cells were analyzed by flow cytometry (Fig. 2). As controls, single cultured unlabeled recipient cells, CMTPX labeled recipient cells (Fig. 2a) or eFluor670 labeled recipient cells (Fig. 2b) and Pgp-donor cells were used. In both experiments Pgp-EGFP was transferred to a similar extent to recipient cells (CMPTX about 6% and eFluor670 about 8% of the hCMEC/D3 wildtype cells).

### Transferred Pgp is functional

**Figure 3:**
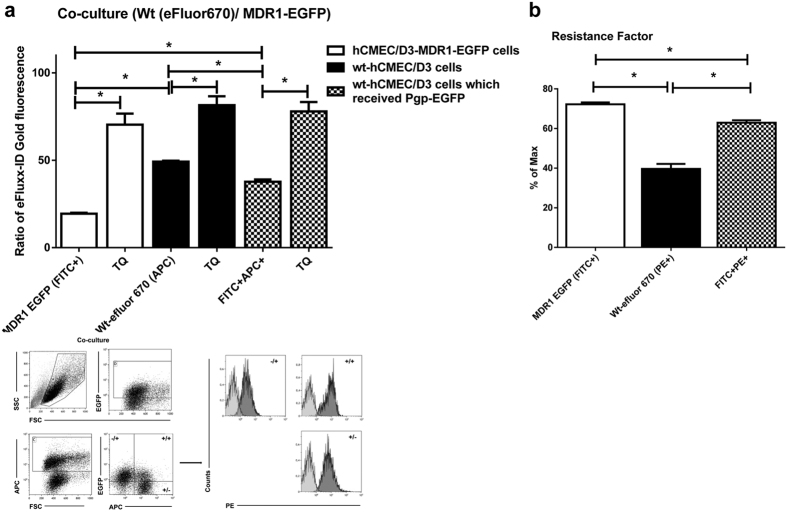
Intercellular Pgp transfer is functional. (**a**) To characterize if transferred Pgp-EGFP in co-cultures is functional, the efflux of the Pgp substrate eFLUXX-ID Gold was measured by flow cytometry. Therefore hCMEC/D3-MDR1-EGFP cells (Pgp-donor cells) were co-cultured with an equal amount of eFluor670-labeled hCMEC/D3 cells (Pgp-recipient cells). After 48 hours of co-culturing three different cell types were identified by flow cytometry in the co-culture: 1. hCMEC/D3-MDR1-EGFP cells (open columns), 2. eFluor670-labeled wt-hCMEC/D3 cells (black columns), and 3. eFluor670-labeled wt-hCMEC/D3 cells which received the fusion protein Pgp-EGFP (hatched columns). For these three cell types present in the co-culture, alterations in Pgp efflux were indirectly measured by determining intracellular concentration of the Pgp substrate eFLUXX-ID Gold for each individual cell. The same experiments were performed in the presence of the Pgp-inhibitor tariquidar (TQ). Significant differences between the three different cell types in the co-culture in intracellular eFLUXX-ID Gold fluorescence are indicated by asterisk (P < 0.05). Tariquidar significantly increased the intracellular accumulation of eFLUXX-ID Gold and its fluorescent metabolite in all cells without inter-group differences, indicating maximal Pgp inhibition in all cell types. As expected, in the absence of tariquidar, the Pgp activity in the co-cultured hCMEC/D3-MDR1-EGFP cells (open columns) was significantly higher compared to co-cultured eFluor670- labeled wt-hCMEC/D3 cells (black columns). Transferred Pgp-EGFP was functional because eFluor670-labeled hCMEC/D3 cells, which received the fusion protein Pgp-EGFP (hatched columns), showed significantly increased Pgp activity compared to eFluor670 labeled wt-hCMEC/D3 cells (black columns) in the absence of Pgp inhibition. (**b**) Assessment of the multidrug resistance factor according to Huber *et al.*^24^. A 1.5-fold increase in the Pgp activity is observed in the hCMEC/D3 cells that have been co-cultured with hCMEC/D3-MDR1-EGFP cells as compared to wt-hCMEC/D3 cells. The multidrug resistance factor was calculated as described in Methods. Significant differences of intracellular eFLUXX-ID Gold fluorescence are indicated by asterisk (P < 0.05).

We next addressed the question whether the transferred Pgp-EGFP fusion protein is functional. For this purpose, we performed flow cytometry experiments with the fluorescent Pgp substrate eFLUXX-ID Gold, which allows multiplexing with EGFP-expressing cell lines and has been used as an indicator of a multidrug resistant phenotype^21^,^23^,^24^,^31^,^32^. Furthermore, the Pgp inhibitors tariquidar and verapamil were utilized to quantitatively measure functionality of Pgp in the co-cultures of hCMEC/D3-MDR1-EGFP (−/+) and eFluor670 labeled hCMEC/D3 cells (+/−) as well as eFluor670 labeled hCMEC/D3 cells which received the Pgp-EGFP fusion protein (+/+) (Figs 3a,b and S2).

Co-cultured hCMEC/D3-MDR1-EGFP (donor) cells that were not treated with tariquidar or verapamil showed a markedly reduced intracellular fluorescence of eFluxx ID-Gold as compared to hCMEC/D3 wildtype cells (Figs 3a and S2), indicating that the Pgp-EGFP fusion protein was functional in the co-cultures. Consistently, hCMEC/D3 recipient cells to which Pgp-EGFP was transferred intercellularly showed an increased Pgp activity as compared to hCMEC/D3 wildtype cells in the co-cultures (Figs 3a and S2).

As indicated by the multidrug resistance factor (MAF), which also includes the response of cells to a Pgp inhibitor (see Methods), Pgp activity of hCMEC/D3-MDR1-EGFP cells was almost 2-fold higher as compared to hCMEC/D3 wildtype cells (Figs 3b and S2). HCMEC/D3 cells to which Pgp-EGFP was transferred revealed a significant elevation of Pgp activity by a factor of 1.5 in comparison to hCMEC/D3 wldtype cells (Figs 3b and S2).

To exclude potential artifacts that may arise from the intercellular transfer and activity of the fusion protein Pgp-EGFP, we performed co-culture experiments with doxycycline-inducible hCMEC/D3-EGFP cells. In contrast to the markedly increased Pgp activity of Pgp-EGFP recipient wild type cells shown in Fig. 3, we observed only a slight decrease in Pgp activity in cells to which EGFP was transferred as compared to hCMEC/D3 wildtype cells (Fig. S3).

### HDAC inhibitors increase intercellular Pgp transfer

**Figure 4:**
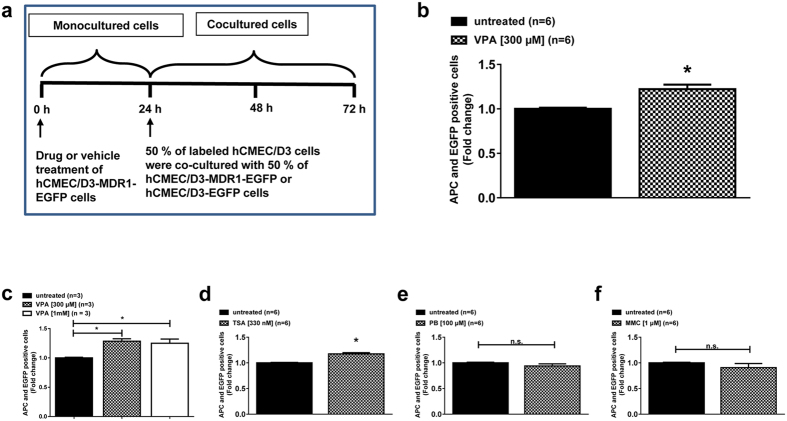
HDAC inhibitors increase cell-to-cell Pgp transfer. (**a**) Schematic illustration of the drug experiments. HCMEC/D3-MDR1-EGFP cells (Pgp-donor cells) were treated with the antiepileptic drugs VPA (**b**) and PB (**e**) for 24 h and then co-cultured with an equivalent cell number of untreated eFluor670 labeled hCMEC/D3 cells (Pgp-recipient cells). To prove the consistency and dose-dependency of VPA’s effect, the experiment was repeated (**c**). Since HDAC inhibition might be involved in the effects of VPA, we exposed cells to the HDAC inhibitor TSA (**d**) and as a further control the intracellular Pgp-trafficking inducing substance MMC (**f**). Flow cytometry experiments revealed increased Pgp-EGFP transfers upon treatment of HDAC inhibitors VPA (**b,c**) and TSA (**d**) to a comparable extent. Three to six experiments were performed per drug with the measurement of 10,000 individual cells per experiment. Significant differences of intracellular eFLUXX-ID Gold fluorescence are indicated by asterisk (P < 0.05).

To investigate whether AEDs known to induce Pgp also modulate intercellular Pgp-EGFP transfer in human brain endothelial cells, single cultured Pgp-donor cells were treated with either VPA or PB for 24 hours and then co-cultured with an equivalent cell number of untreated eFluor670 labeled Pgp-recipient cells for further 48 hours (Fig. 4a). We observed significantly increased Pgp-EGFP transfer upon VPA treatment (Fig. 4b,c) whereas such increase was not observed for PB treatment (Fig. 4e).

To test whether inhibition of HDACs by VPA may be involved in the increased Pgp-EGFP transfer, we treated Pgp-donor cells with the specific HDAC inhibitor TSA and observed significant induction of Pgp-EGFP transfer (Fig. 4d). An increase in the VPA concentration from 300 μM to 1 mM did not further enhance its effect (Fig. 4c). An increase in TSA concentrations from 330 nM to 1 μM induced cell death of the treated cells in the co-culture experiments, which was not observed with any other drug. Treatment of cells with MMC, which we recently found to increase intracellular trafficking of Pgp^21^, did not increase intercellular Pgp transfer (Fig. 4f).

**Figure 5:**
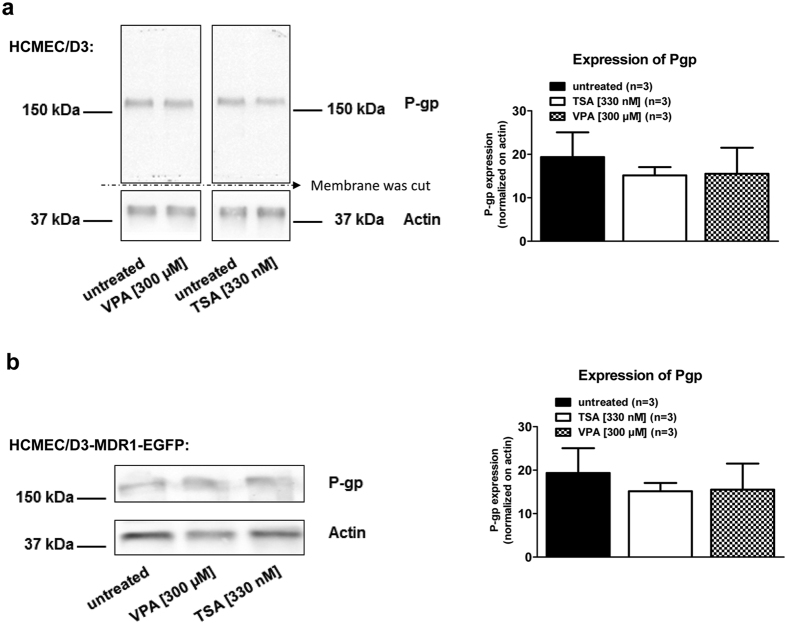
VPA and TSA do not increase the expression of Pgp or Pgp-EGFP. Expression of endogenous Pgp in wt-hCMEC/D3 cells or hCMEC/D3-MDR1-EGFP cells (in the absence of doxycycline) was not modified by treatment with the HDAC inhibitors VPA and TSA. HCMEC/D3 cells (**a**) or hCMEC/D3-MDR1-EGFP cells (**b**) were treated with VPA (300 μM) or the HDAC inhibitor TSA (330 nM) for 24 hours. Detergent extracts were analyzed by Western blotting using anti-GFP antibodies to detect GFP-tagged Pgp and anti-actin. Pgp and actin bands of three independent experiments were analyzed densitometrically and Pgp signals were normalized versus actin. It should be noted that different gels were used for the experiments with hCMEC/D3 and hCMEC/D3-MDR1-EGFP cells, so that small differences in protein migration may be missed. When both cell types were run on the same gel, hCMEC/D3 wild type cells expressed a 170-kDa protein band corresponding to endogenous Pgp whereas non-induced hCMEC/D3-MDR1-EGFP cells expressed an approximately 200-kDa protein band corresponding to the predicted size of the chimeric MDR1-EGFP^21^.

**Figure 6:**
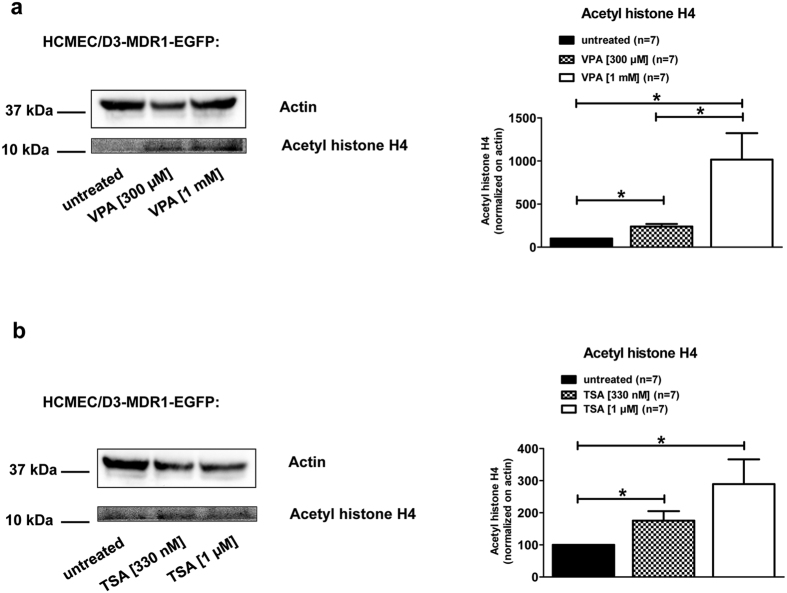
VPA and TSA increase acetylation of histone H4 in hCMEC/D3 cells. Western blot studies revealed an increased acetylation of histone H4 in a concentration-dependent manner upon VPA (a; 300 μM and 1 mM) and TSA (**b**; 330 nM and 1 μM) treatment. In these experiments, hCMEC/D3-MDR1-EGFP cells (Pgp-donor cells) were treated for 24 hours with 300 μM or 1 mM VPA (**a**) or 330 nM and 1 μM of the HDAC inhibitor TSA (**b**). Acetyl histone H4 and actin bands of seven experiments were analyzed densitometrically and Pgp signals were normalized to actin. Significant differences of three independent experiments were analyzed densitometrically and acetylations of histone H4 were normalized versus actin.

For further exploring the mechanism of the effect of VPA, Western blot analysis was performed prior to co-cultivation to determine the protein levels of endogenous Pgp in hCMEC/D3 cells, Pgp-EGFP fusion protein and the acetylation of histone H4 in hCMEC/D3-MDR1-EGFP cells upon VPA and TSA treatments (Figs 5 and 6). Neither the endogenous expression of Pgp in hCMEC/D3 cells (Fig. 5a) nor the amount of Pgp-EGFP fusion protein in hCMEC/D3-MDR1-EGFP cells (Fig. 5b) were affected by VPA (300 μM) or TSA (330 nM) exposure for 24 hours. Acetylation of histone H4 was increased in a concentration-dependent manner upon VPA treatment (300 μM and 1 mM, Fig. 6a) as well as upon TSA treatment (330 nM and 1 μM, Fig. 6b).

## Discussion

The current communication reveals a novel trafficking pathway of Pgp between brain capillary endothelial cells. These findings have important implications for BBB function and resistance to therapy, since Pgp along this pathway retains its function in the recipient cells and confers an increased resistance to Pgp substrates on the recipient cell. We further demonstrate that the HDAC inhibitors VPA and TSA increase intercellular Pgp transfer.

Intercellular transfer of Pgp has been first reported in 2005 in tumor cells^2^ and various studies since then have confirmed and extended this finding to several cancer cell lines^13^. Levchenko *et al.*^2^ proposed this protein transfer to be the result of a process requiring either cell-to-cell contact or exchange of relatively large membrane microparticles. Subsequent studies indicated that intercellular transfer of Pgp-containing microvesicles is a main process underlying the transfer of Pgp from donor to recipient cancer cells^13^,^33^. In the present study, we observed that cell-to-cell contact between adherent Pgp donor and recipient cells leads to the exchange of Pgp-containing vesicles in the human brain capillary endothelial cell line hCMEC/D3, which is widely used as a model of the human BBB^34^. Intercellular Pgp transfer was associated with a significantly enhanced efflux activity in the Pgp recipient cells as measured by efflux of a Pgp substrate.

When the Pgp donor cells were exposed to drugs before co-culturing with recipient cells, a significant increase in intercellular Pgp transfer was observed with the HDAC inhibitors VPA and TSA, while PB or MMC exposure did not affect this process. At the concentrations used, both VPA and TSA induced a significant increase in acetylation of histone H4 in the Pgp donor cells, thus suggesting that the increased Pgp transfer may be a result of HDAC inhibition. In contrast to the increased intercellular Pgp trafficking, VPA and TSA did not induce the expression of endogenous Pgp in donor or recipient cells, as reported previously for VPA and hCMEC/D3 cells^27^. In cancer cells, VPA and other HDAC inhibitors have been reported to increase Pgp expression, although the results for VPA are equivocal^35^. This may be a result of differences in cell type, drug concentration, or duration of drug exposure. For instance, in the study of Eyal *et al.*^26^ in human cancer cell lines and rat liver, significant effects on Pgp expression were only observed at higher VPA concentrations and longer VPA exposure than used in the present experiments. In this respect, it is important to note that the VPA concentration used in the present study (300 μM) is within the therapeutic plasma concentration range of VPA in patients with epilepsy^36^.

The effect of VPA on acetylation of histone H4 was concentration-dependent with a 2.4-fold increase at 300 μM and a 10-fold increase at 1 mM, whereas the increase in intercellular Pgp transfer by VPA reached its maximum of 22–28% at 300 μM and not further increased at 1 mM. This is most likely a result of the model characteristics used for studying intercellular Pgp transfer (Fig. 4a) which may limit the maximum increase that can be obtained with HDAC inhibitors.

How may HDAC inhibition induce intercellular Pgp transfer? Initially regarded as epigenetic modifiers that act predominantly through chromatin remodeling via histone acetylation, HDAC inhibitors have since been recognized to be truly pleiotropic agents which act through a wide variety of disparate and mutually interactive mechanisms, including signal transduction^37^,^38^. One interesting target in this respect are rab GTPases, which are known to participate in intracellular pathways of Pgp^39^ and transcellular vesicle-mediated transport of nanoparticles^40^. Furthermore, components of the cytoskeleton, which are regulated by HDACs, may be also involved^11^,^37^.

There are several paths a “naive” BBB endothelial cell can take to increase the expression of Pgp^41^. The intrinsic expression of Pgp in BBB endothelial cells is relatively high, which contributes to the barrier function, restricting brain entry of numerous small lipophilic compounds, including anticancer drugs, which otherwise would penetrate through the BBB without any limitation^14^. Known paths through which Pgp expression at the BBB can be further enhanced include polymorphisms in *MDR1* (*ABCB1*) and upregulation of Pgp expression by inflammation, seizures (through neuronal release of glutamate), and various therapeutic drugs and environmental toxicants (through ligand-activated transcription factors); as a consequence of increased BBB Pgp, drug delivery to the brain is reduced^41^,^42^. Furthermore, we recently demonstrated drug-induced intracellular trafficking of Pgp between intracellular sites and the plasma membrane in human BBB endothelial cells, which was regulated by lipid rafts^21^. The present demonstration of intercellular Pgp trafficking between adherent human BBB endothelial cells and its modulation by HDACs adds to the complexity of Pgp regulation at the BBB.

The increase of Pgp cell-to-cell transfer by the anti-seizure drug VPA in a BBB model may be important to further understand the mechanisms involved in multidrug resistance (MDR) of epilepsy patients to AEDs, which occurs in about 30% of all cases^17^. Although VPA seems not to be a substrate of Pgp^19^, increase in BBB expression of Pgp by VPA would enhance the brain efflux of other AEDs that are Pgp substrates^43^. In addition to the role of the BBB in epilepsy^14^,^20^,^44^, the increased intercellular transfer of Pgp found with VPA and TSA in this study may be an important aspect in the treatment of cancer by these HDAC inhibitors. VPA, TSA and several other HDAC inhibitors currently undergo clinical trials for treatment of different types of cancer, and two HDAC inhibitors, vorinostat and romidepsin, have been approved by the Food and Drug Administration to treat T-cell lymphoma^35^,^45^. The interest in HDAC inhibitors as potent anticancer drugs is due to their broad anti-tumor activity and low toxicity in normal cells^45^. However, as with other categories of anticancer drugs, treatment of cancer with HDAC inhibitors can lead to broad-spectrum anticancer MDR, resulting in cells that are resistant to numerous structurally and functionally unrelated drugs^35^. One of the phenotypes of MDR is the upregulation of ABC transporters, such as Pgp, which decreases the level of intracellular chemotherapeutic drugs in an energy-dependent manner^35^. The present data indicate that HDAC inhibitor effects on intercellular trafficking of Pgp and other efflux transporters should be studied as a potential mechanism of MDR in cancer cells, too.

One potential problem of the present data and their interpretation is that the experiments were performed in culture with immortalized brain capillary endothelial cells that, although they express tight junction proteins, do not reach the high transendothelial electric resistance resistance (TEER) and low flux of paracellular permeability markers that is characteristic for BBB models using primary cultures of such cells^22^,^46^. We would expect that higher expression of tight junction proteins and the resultant enhancement of TEER (reflecting barrier tightness) would result in enhanced intercellular transfer of Pgp, because we assume that cell-to-cell contact between adherent cells is the major mechanism of Pgp transfer. We plan to study this by pharmacological increase of tight junction proteins in hCMEC/D3 cells^47^, co-culturing with astrocytes and pericytes^48^, as well as by using a human BBB model derived from hematopoietic stem cells^49^.

In conclusion, the drug efflux pump Pgp can be transferred from Pgp-overexpressing cells to cells with low Pgp expression in a human model of the BBB, and this process is enhanced by HDAC inhibitors. Non-genetic transfer of a resistance phenotype is a novel mechanism of alterations in BBB functionality. The clinical significance of this mechanism will depend on whether transfer of Pgp at critical levels occurs *in vivo*. We plan to study this question by positron emission tomography (PET) and ^11^C-labelled Pgp substrates such as (*R*)-[^11^C]-verapamil^50^, following treatment with and without HDAC inhibitors. As shown previously, the advantage of this strategy is that it can be rapidly translated from experimental animals to humans^51^,^52^.

## Additional Information

**How to cite this article**: Noack, A. *et al.* Intercellular transfer of P-glycoprotein in human blood-brain barrier endothelial cells is increased by histone deacetylase inhibitors. *Sci. Rep.* **6**, 29253; doi: 10.1038/srep29253 (2016).

## Supplementary Material

Supplementary Information

Supplementary Information
