# Supplementary material for: Intercellular transfer of P-glycoprotein in human blood-brain barrier endothelial cells is increased by histone deacetylase inhibitors
Source: Sci Rep. 2016 Jul 4;6:29253. doi: 10.1038/srep29253 (PMC4931680; doi:10.1038/srep29253)
Supplement: Supplementary Information [file srep29253-s1.pdf]

Intercellular transfer of P-glycoprotein in human blood-brain barrier endothelial cells is increased by histone deacetylase inhibitors

Andreas Noack, Sandra Noack, Manuela Buettner, Hassan Y. Naim, and Wolfgang Löscher

Supplementary Information

Supplementary Video S1. Intercellular Pgp trafficking is mediated by vesicles. The video shows a period of 48 hours in fast motion of co-cultured hCMEC/D3 recipient cells and hCMEC/D3-MDR1-EGFP donor cells. Images were taken in an incubator with an inverted fluorescence microscope. Red arrows show intercellular Pgp-EGFP transfer by vesicular structures and the orange arrow an extracellular Pgp-EGFP vesicle in the supernatant of the cells. The fusion protein Pgp-EGFP is shown in green.

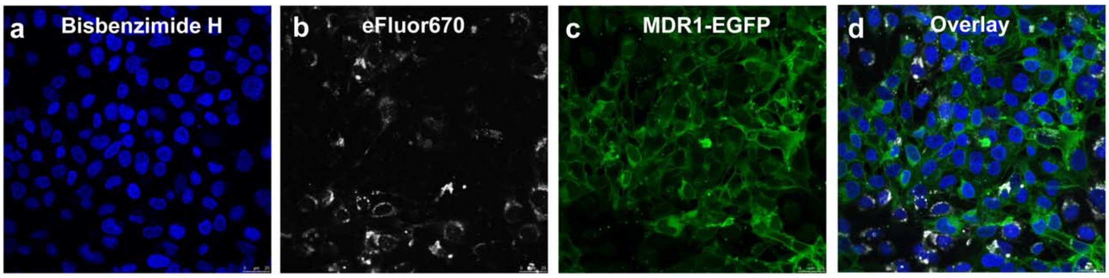

Supplementary Figure S1: Illustration of the three staining methods used to demonstrate intercellular Pgp-EGFP transfer as illustrated in Fig. 1b. (a) shows staining of a co-culture (50:50) of Pgp-recipient (hCMEC/d3) and Pgp-donor (hCMEC/D3-MDR1-EGFP) cells stained with bisbenzimide H (as a fluorescent stain for DNA); (b) shows the same co-culture in which Pgp-recipient (wildtype) cells were stained with the cell tracker eFluor670 before co-culturing with Pgp-donor (hCMEC/D3-MDR1-EGFP) cells; (c) shows the green (EGFP) fluorescence of the Pgp-donor cells; and (d) illustrates the overlay of a, b, and c. (b) clearly shows that the cell tracker (eFluor670) used in these experiments is not transferred between Pgp-recipient and Pgp-donor cells in the co-culture, excluding that just the dye is being transferred, not the Pgp. This is also demonstrated in Video S1, which demonstrates Pgp transfer in the absence of any cell tracker.

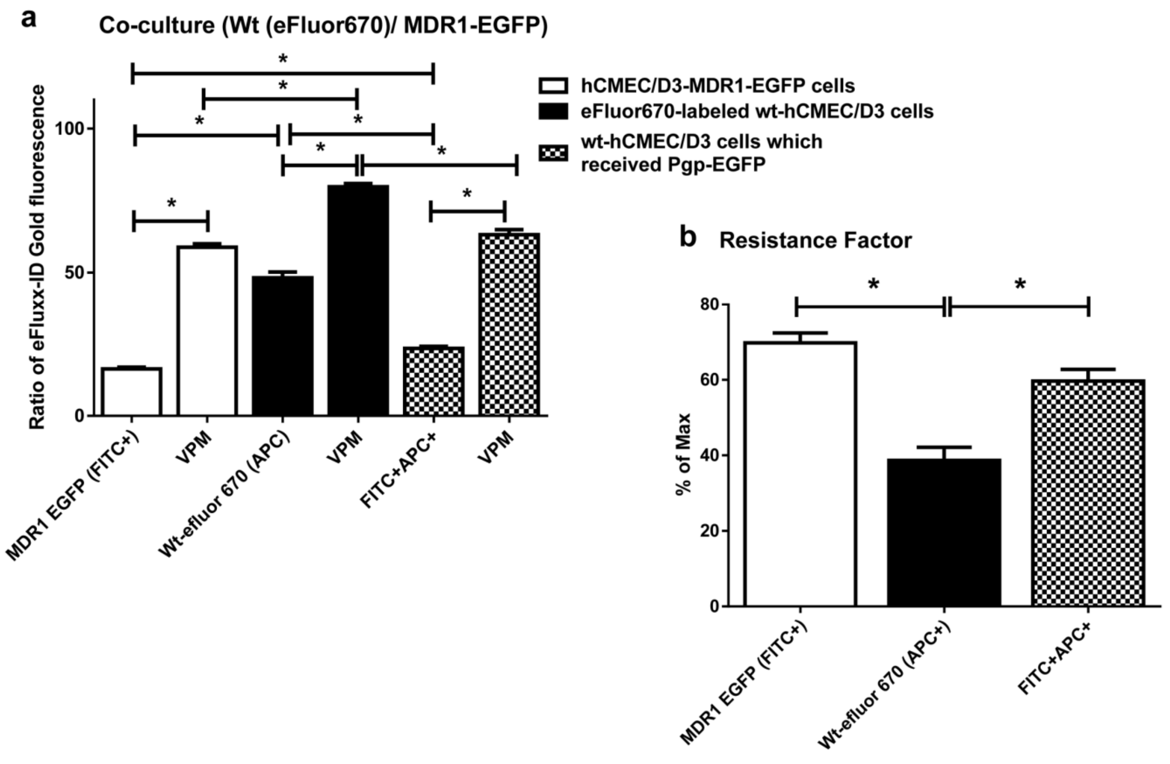

Supplementary Figure S2: Intercellular Pgp transfer is functional. (a) Flow cytometry experiments were performed to characterize if transferred Pgp-EGFP in co-cultures is functional. As described in detail in the legend of Fig. 3, the efflux of the Pgp substrate eFLUXX-ID Gold was measured by flow cytometry. The only difference between Fig. 3 and Supp. Fig. 1 is that experiments were performed in the presence of 20 µM of the Pgp inhibitor verapamil instead of tariquidar. Similar effects were observed as in Fig. 3. In the absence of verapamil, the Pgp activity in the co-cultured hCMEC/D3-MDR1-EGFP cells (open columns) was significantly increased (by 100%) compared to co-cultured eFluor670- labeled wt-hCMEC/D3 cells (black columns). Significant differences of intracellular eFLUXX-ID Gold fluorescence are indicated by asterisk ($P < 0.05$).

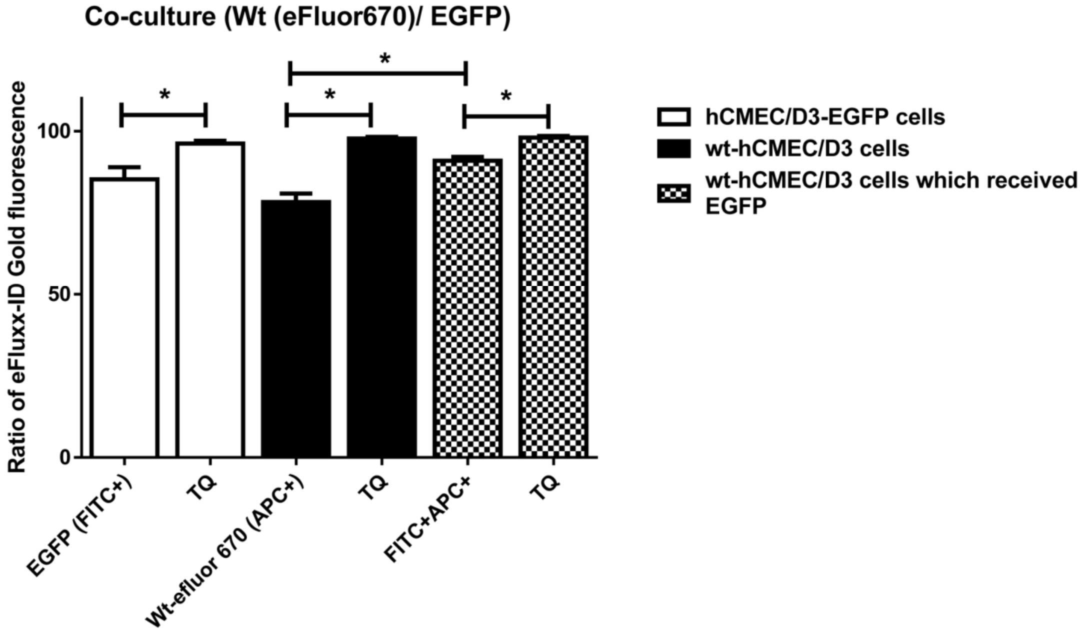

Supplementary Figure S3: Transfer of EGFP from hCMEC/D3-EGFP cells to hCMEC/D3 cells decreases Pgp activity. To control for unspecific effects by expression and transfer of the Pgp-EGFP fusion protein we generated a hCMEC/D3-EGFP cell line and used it in co-culture experiments in combination with the functional eFLUXX ID-Gold assay. Flow cytometry analysis revealed that the EGFP transfer does not increase but rather decrease Pgp activity in eFluor670 labeled hCMEC/D3 cells. Intracellular accumulation of eFLUXX-ID Gold was measured in tariquidar-treated or untreated co-cultures. Experiments were performed in triplicate with the measurement of 10,000 individual cells. Significant differences of intracellular eFLUXX-ID Gold fluorescence are indicated by asterisk ($P < 0.05$). Note the low efflux of eFLUXX-ID Gold in the hCMEC/D3-EGFP cells (left open column), which is obviously due to endogenous Pgp, compared to the marked efflux of eFLUXX-ID Gold in hCMEC/D3-MDR1-EGFP cells as illustrated in Fig. 3A (left open column). These data substantiate that under the conditions of these experiments, eFLUX-ID

Gold is a selective substrate of Pgp.
